# Nomogram for Individualized Prediction of Hepatocellular Carcinoma with Portal Vein Tumor Thrombosis on Conservative Treatment

**DOI:** 10.1155/2020/1473718

**Published:** 2020-02-17

**Authors:** Yao Liu, Dongying Xue, Xiaogang Zhang, Fangyuan Gao, Le Sun, Xue Yang, Yuxin Li, Qun Zhang, Bingbing Zhu, Shuaishuai Niu, Yunyi Huang, Ying Hu, Xianbo Wang

**Affiliations:** ^1^Center of Integrative Medicine, Beijing Ditan Hospital, Capital Medical University, Beijing 100015, China; ^2^Department of Infections Disease, Putuo Hospital, Shanghai University of Traditional Chinese Medicine, Shanghai 200062, China; ^3^Institute of Liver Diseases, Third People's Hospital of Changzhou, Changzhou 213000, China; ^4^Department of Gastroenterology, Dongzhimen Hospital, Beijing University of Chinese Medicine, Beijing 100700, China

## Abstract

**Background:**

Portal vein tumor thrombosis (PVTT) is one of the major predictive factors for patients with hepatocellular carcinoma (HCC). The objective of this study was to establish a prognostic nomogram for identifying individual survival outcomes in patients with HCC and PVTT on conservative treatment based on specific factors.

**Methods:**

Two hundred and ten patients with HCC and PVTT on conservative treatment in Beijing Ditan Hospital between June 2008 and May 2017 were studied retrospectively as a derivation cohort. We built a nomogram based on independent risk factors for survival prediction. The concordance index (c-index) and a calibration curve were used to evaluate the predictive accuracy. During the study, 102 patients were included at the Putuo Hospital and Third People's Hospital of Changzhou as a validation cohort.

**Results:**

In the derivation cohort, the independent factors for overall survival were identified by multivariate analysis, namely, aspartate aminotransferase ≥119 IU/L, gamma-glutamyl transferase ≥115 IU/L, Child–Pugh class C liver function, creatinine ≥91 *μ*moI/L, *α*-fetoprotein ≥400 ng/ml, and largest tumor diameter ≥5 cm. The nomogram had a c-index of 0.737 (95% confidence interval, 0.692–0.782) and the calibration curves fitted well. The median survival time was 4.2 months in the derivation cohort, with an MST of 5 months for BCLC C stage and 1.8 months for BCLC D stage patients. Kaplan–Meier analysis showed significant statistical differences in the 6-month overall survival rates of the primary and validation cohorts after the total scores were divided into three quartiles (low risk: 0–85; intermediate risk: 86–210; high risk: ≥211; *p* < 0.0001 in both cohorts).

**Conclusions:**

The nomogram can be a more accurate and individualized prediction for 6-month overall survival of patients with HCC and PVTT on conservative treatment, and it is possible to consider further active interventions for patients in the low-risk group (0–85 scores) to achieve the aim of prolonging survival.

## 1. Introduction

Hepatocellular carcinoma (HCC) is typically developing in individuals with chronic liver disease or cirrhosis [1]. HCC is characterized by its propensity to invade the vasculature within the liver [2]. Portal vein tumor thrombosis (PVTT) is a common phenomenon in unresectable HCC, which is observed in 10–60% of HCC patients [3–5]. PVTT can cause intrahepatic or distant metastasis through the portal system and lead to liver dysfunction and portal hypertension, leading to ascites formation, variceal rupture, hepatic encephalopathy, and/or death [6]. PVTT is an independent predictor of tumor recurrence and is widely accepted as a sign of advanced stage, which frequently leads to a poor prognosis [7, 8]. PVTT has categorized into five grades: Vp0, no PVTT; Vp1, distal to the second-order branches; Vp2, in the second-order branches; Vp3, in the right first-order branches; and Vp4, in the main trunk or left first-order branches (or both) (The Liver Cancer Study Group of Japan) [9].

HCC associated with PVTT is considered an advanced stage with no curative therapy according to the Barcelona Clinic for Liver Cancer (BCLC) staging system and treatment guidelines [10]. Palliative sorafenib chemotherapy is the only therapeutic method for this group of patients [4]. However, studies have shown that the median survival time is only 8.1 months in patients treated with sorafenib [11]. Except for the sorafenib treatment recommended by BCLC, radiofrequency, locoregional therapy, and surgical resection may improve the prognosis of patients with HCC and PVTT [12]. Unfortunately, the median survival duration after these treatments is still unsatisfactory.

As mentioned above, various attempts have been made to treat HCC with PVTT. However, transcatheter arterial chemoembolization (TACE) is contraindicated for patients with main PVTT because of the potential risk of acute liver failure or infarction due to ischemia after the procedure. Furthermore, patients with end-stage liver disease including severely impaired liver function (Child–Pugh C) and/or severe complications such as massive ascites, hepatic encephalopathy, upper gastrointestinal bleeding, or poor Eastern Cooperative Oncology Group performance status (ECOG PS) cannot receive surgical resection or locoregional therapy, but can only receive symptomatic supportive treatment [13, 14]. This study was based on the use of symptomatic supportive care for patients who were not eligible for the surgical resection, locoregional therapy, or sorafenib chemotherapy.

Although some staging models are applied to classify tumors for selecting suitable treatment modalities, such as BCLC staging [15], tumor, node, metastasis (TNM) staging (the American Joint Committee on Cancer 2010) [16], Cancer of the Liver Italian Program, Japanese Integrated Staging, and Okuda staging systems, these models do not assess the survival prognosis of individuals with HCC and PVTT. But individualized predicting or stratifying patients of HCC associated with PVTT on conservative treatment remain unsatisfactory. The prognostic nomograms can be more accurate and allow personalized assessment of overall survival for patients with HCC [17]. The purpose of the present study was to develop a nomogram based on specific factors for predicting individual survival outcomes in HCC patients with PVTT on conservative treatment.

## 2. Materials and Methods

### 2.1. Patient Selection

Conservative therapy is recommended in patients with massive ascites, upper gastrointestinal bleeding due to esophageal varices, hepatic encephalopathy, Child–Pugh class C liver function, and poor ECOG PS and those who did not suitable for sorafenib treatment. Patients with HCC and PVTT who underwent conservative therapy did not receive local or systemic chemotherapy, radiotherapy, radiofrequency ablation, and/or surgical resection. Instead, they received symptomatic supportive care mainly to treat complications, such as ascites, hypersplenism, upper gastrointestinal bleeding, hepatorenal syndrome, and hepatic failure caused by portal hypertension.

The clinical data of all patients with HCC and PVTT on conservative treatment were analyzed retrospectively. A total of 210 patients were included in the derivation cohorts in Beijing Ditan Hospital (Beijing, China), Capital Medical University, between October 2008 and May 2017. 102 patients were enrolled in the validation cohorts in Putuo Hospital (Shanghai, China), Shanghai University of Traditional Chinese Medicine, and Third People's Hospital of Changzhou (Changzhou, China) between October 2010 and September 2016. HCC was diagnosed based on the recommendations of the European Association for the Study of Liver Disease [18] or American Association for the Study of Liver Diseases [13], which included a serum alpha-fetoprotein (AFP), ultrasound, computed tomography (CT), magnetic resonance imaging (MRI), and angiography. PVTT showed portal vein filling defect in contrast-enhanced imaging (CT-scan or MRI), and embolic enhancement was identical or similar to that observed in patients with primary liver cancer [19]. Patients with autoimmune liver disease, hepatitis A, D, or E, syphilis, or acquired immune deficiency syndrome and patients with other primary malignancies were excluded. Patients were not included in the study if the data were incomplete or not available for follow-up. This project was approved by the Ethics Committee of Beijing Ditan Hospital (Beijing, China).

### 2.2. Data Collection

Clinical data included the demographic status (age and gender), etiology (HBV-Ag, anti-HCV, and alcohol consumption), complications (ascites, hepatic encephalopathy, and hypersplenism), comorbidity index, liver function (alanine aminotransferase (ALT), aspartate aminotransferase (AST), *γ*-glutamyl transpeptidase (GGT), total bilirubin (TBIL), serum albumin (ALB), and prothrombin time (PT)), blood routine (white blood cell (WBC) count), kidney function (creatinine (Cr)), and tumor-related indexes (*α*-fetoprotein (AFP), tumor number, largest tumor diameter, and lymph node metastasis). The data were collected at the time of diagnosis of HCC with PVTT.

To investigate the effect of the complications on prognosis, we recorded the comorbidities of patients and calculated the Deyo comorbidity index, which is an adjustment of the Charlson disease severity index [20]. The index included 11 categories of diseases as follows: myocardial infarction, congestive heart failure, peripheral vascular disease, cerebrovascular disease, dementia, chronic pulmonary disease, rheumatic disease, peptic ulcer disease, diabetes, hemiplegia or paraplegia, and renal disease. We did not include mild, moderate, or severe liver disease, any malignancy, metastatic solid tumor, or AIDS as they did not meet the characteristics of this study. This score was included as a covariate in Cox regression analysis.

### 2.3. Follow-Up

Patient survival was the endpoint of the study, which was measured in months from the time of the initial diagnoses of HCC with PVTT to death or the last follow-up date. The number of days of survival for each patient was obtained when possible, and then divided the number of days/30 for the exact number of months. Therefore, the patient was not available for follow-up and not included in the study.

### 2.4. Statistical Analysis

Demographic and clinical characteristics were summarized as the median and range or number. Most experimental data have been converted to categorical variables based on cutoff values, which were calculated based on the maximum Youden index (sensitivity + specificity − 1) values. Categorical variables were detected using the chi-square test to compare the differences between two groups. Detection of continuous variables conforming to normal distribution and variance homogeneity was determined using the *t*-test. Overall survival (OS) was analyzed using the Kaplan–Meier method, which was calculated from the date of diagnosis of HCC with PVTT to death or the last follow-up time. Univariate and multivariate Cox proportional hazards regression analyses were carried out to identify significant factors predicting the risk of death. The nomogram was established using independent risk factors affecting prognosis, which is a visual presentation of the Cox regression model. The performance of the nomogram was measured by concordance index (c-index) and assessed by calibration which was performed by bootstrapping. Analyses were performed using SPSS 22.0 statistical package (SPSS, Inc., Chicago, IL, USA) and RMS packages in R version 3.0.2. *p* value < 0.05 was considered statistically significant.

## 3. Results

### 3.1. Characteristics of Derivation and Validation Cohorts

A total of 312 patients diagnosed with HCC with PVTT on conservative treatment were included in this study. Of these patients, the median age was 55 years (range 28–91 years), 274 were male and 38 were female, and the majority were HBV related (83.7%). The most commonly presented complication was ascites (73.3%), followed by hepatic encephalopathy (5.4%). Most subjects were classified as Vp4 PVTT (69.5%), BCLC stage C (81.3%), and TNM stage IIIB (86.9%), had tumors ≥5 cm in diameter (60.6%), had more than three tumors (53.5%), and had no lymph node metastasis (86.2%). The clinical features of the patients in the derivation and validation cohorts are summarized in Table 1.

**Figure 1:**
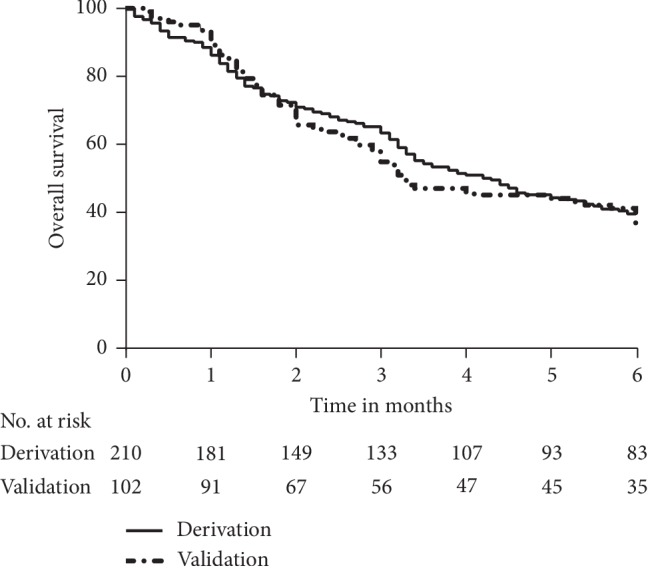
Kaplan–Meier estimates of overall survival in the derivation and validation samples.

Kaplan–Meier estimates of overall survival indicated that the survival rates for derivation samples at 1, 3, and 6 months were 86.2%, 63.3%, and 39.5%, respectively. The survival rates for validation samples at 1, 3, and 6 months were 89.2%, 54.9%, and 34.3%, respectively (Figure 1). The median survival time (MST) was 4.2 months in the derivation cohort, with an MST of 5 months for BCLC C stage and 1.8 months for BCLC D stage patients. The majority of the derivation cohort was classified as Vp3-4 PVTT (92.4%), and 7.6% were classified as Vp2. The MST of Vp3-4 PVTT and BCLC C stage HCC (73.3%) was 5.1 months.

### 3.2. Prognostic Factors of Survival

The results of univariate Cox regression analyses showed that hepatic ALT, AST, GGT, Child–Pugh class, WBC, Cr, AFP, tumor number ≥3, and largest tumor diameter ≥5 cm obtained from the derivation cohort were predictive factors for survival. These factors were included in the multivariate Cox regression analyses. Finally, AST, GGT, Child–Pugh class, Cr, AFP, and largest tumor diameter ≥5 cm were selected as significant factors affecting the prognosis for overall survival by the forward selection procedure (Table 2).

### 3.3. Nomogram

**Figure 2:**
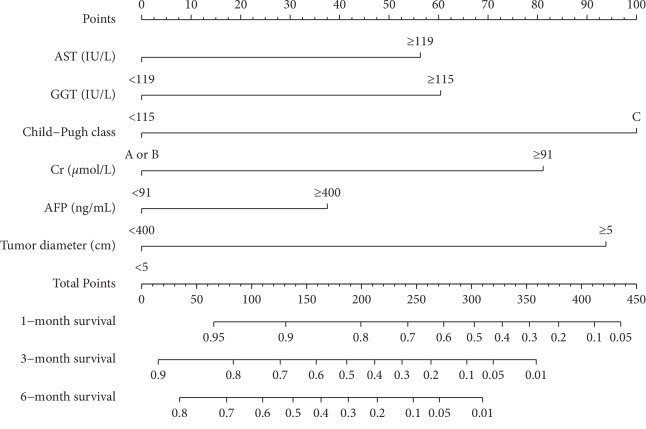
Nomogram predicting 1-, 3-, and 6-month overall survivals of patients with HCC and PVTT on conservative treatment. The total points were obtained by adding the scores of each variable corresponding to the point on the upper pointing axis. A line was then drawn to the probability axis of the graph at the bottom, which indicated the survival probability at 1-, 3, and 6 months.

**Figure 3:**
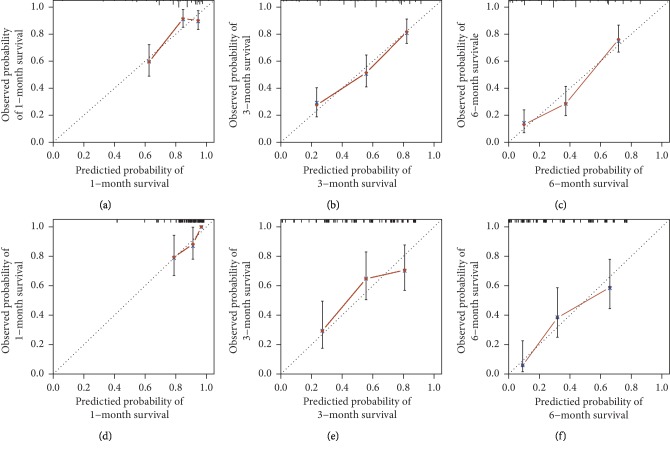
Calibration plot of the nomogram for overall survival in the primary cohort (a–c) and validation cohort (d–f) at 1, 3, and 6 months, in which the predicted probability of survival was compared with the actual survival.

The nomogram was established based on the abovementioned predictors, which were used to predict the survival rates of 1, 3, and 6 months by adding the score corresponding to each factor and projecting the total score to the bottom scale (Figure 2). The c-index for predicting overall survival was 0.737 (95% confidence interval, 0.692–0.782) for the derivation data and 0.709 (95% confidence interval, 0.643–0.775) for the validation data. The calibration curves showed that the predicted survival probabilities were in agreement with the actual results observed in both derivation (Figures 3(a)–3(c)) and validation cohorts (Figures 3(d)–3(f)) for 1-, 3-, and 6-month OS.

### 3.4. Performance of the Nomogram in Stratifying Risk of Patients

**Figure 4:**
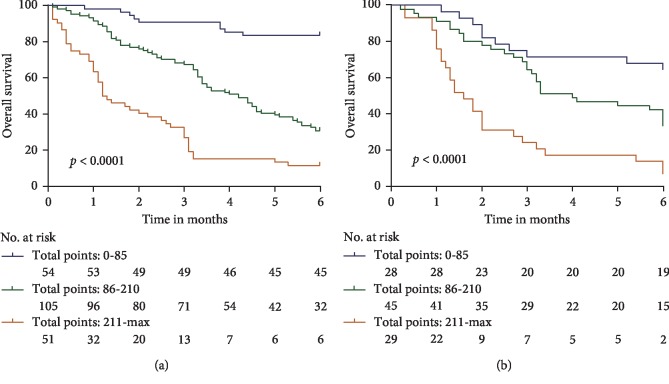
Kaplan–Meier curves of risk group stratification for OS in the derivation (a) and validation cohorts (b). The range of scores for each risk group was as follows: low risk: 0–85, intermediate risk: 86–210, and high risk: ≥211.

Patients were divided into three quartiles according to the total scores: (low risk: 0–85; intermediate risk: 86–210; high risk: **≥**211). Kaplan–Meier analysis showed significant statistical differences in the 1-, 3-, and 6-month OS rates in the primary and validation cohorts after the total scores were divided into three quartiles. 6-month OS rate in the primary cohort is 83.3% in the low-risk group, 30.5% in the intermediate-risk group, and 11.8% in the high-risk group (*p* < 0.0001; Figure 4(a)), and 6-month OS rate in the validation cohort is 67.9% in the low-risk group, 33.3% in the intermediate-risk group, and 6.9% in the high-risk group (*p* < 0.0001; Figure 4(b)).

**Figure 5:**
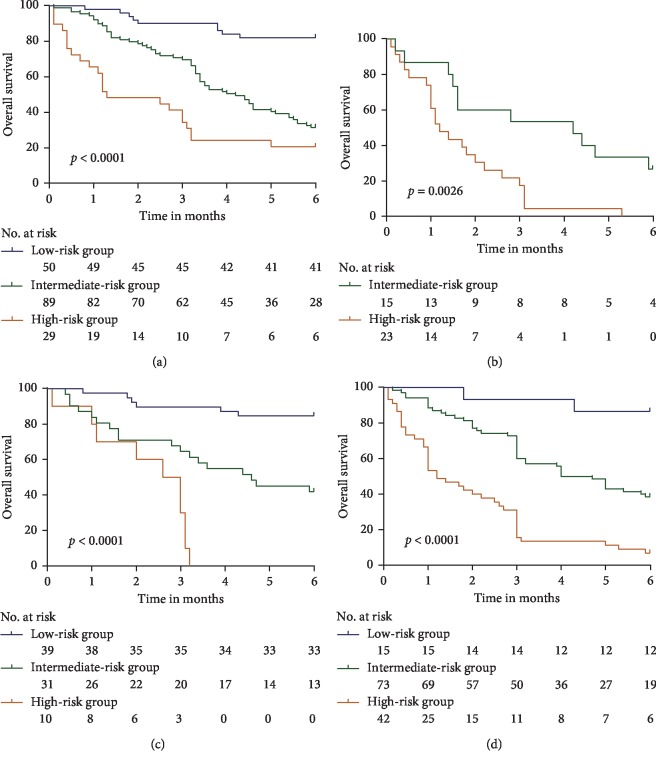
Risk group stratification with tumor characteristics. (a) Subgroup with BCLC C staging, (b) subgroup with BCLC D staging, (c) subgroup with largest tumor diameter <5 cm, and (d) subgroup with largest tumor diameter ≥5 cm. A subgroup of fewer than 10 patients was omitted from the figure.

Furthermore, patients with HCC and PVTT on conservative treatment were stratified for mortality with respect to different tumor characteristics, including BCLC and tumor diameter using a nomogram scoring system. As shown in Figure 5, the Kaplan–Meier curves of the BCLC stages C (Figure 5(a)) and D (Figure 5(b)) and tumor diameter <5 cm (Figure 5(c)) and ≥5 cm (Figure 5(d)) of the primary cohorts indicated that the nomogram stratification showed good prognostic effect.

## 4. Discussion

Despite advances in imaging modalities and improvements in the efficacies of anti-HCC treatment, the incidence of PVTT is increasing. PVTT can induce intrahepatic spread of tumor cells and distal metastasis and can aggravate portal hypertension, resulting in upper gastrointestinal bleeding, the presence of ascites, and liver failure [21, 22]. PVTT is an important cause of poor prognosis in HCC patients [23–26]. At present, several treatments that can prolong the survival time of patients with HCC and PVTT exist; however, with progression of the disease, patients with end-stage liver cancer usually cannot undergo any intervention, but can only receive support treatment. Therefore, it is necessary to accurately predict individual survival outcomes in HCC patients with PVTT on conservative treatment according to clinical characteristics. According to our research, the mortality rate for HCC with PVTT on conservative treatment increased with an increase in the follow-up period. This resulted in the mortality rate reaching 84.2% in 12 months, making the evaluation of overall survival insignificant. In addition, the median survival time for HCC with PVTT patients was only 2.7–4 months [27]. Therefore, the 6-month overall survival evaluation was valuable. In the present study, the clinical and radiologic characteristics of HCC patients with PVTT were incorporated into multivariate Cox regression analyses. We found that AST ≥119 IU/L, GGT ≥115 IU/L, Child–Pugh class C liver function, Cr ≥ 91 *μ*moI/L, AFP ≥400 ng/ml, and largest tumor diameter ≥5 cm were independent predictors of individual survival outcomes. Based on these six parameters, an effective and easy-to-apply nomogram was set up to estimate the individual survival prognosis of patients with HCC with PVTT on conservative treatment at 1, 3, and 6 months.

Previously suggested prognostic indices for PVTT, including neutrophil-lymphocyte ratio (NLR), ascites, arteriovenous fistula, and TACE response [28, 29], are associated with individual survival outcomes. A study by Jeong et al. reported that the tumor size, tumor diversity, PVTT site, degree of portal vein occlusion, lymph node metastasis, Child–Pugh grading, and the Eastern Cooperative Oncology Group performance status are favorable prognostic factors for the survival of HCC patients with PVTT on radiotherapy. They also reported that their scoring system showed better prognostic ability than the Cancer of the Liver Italian Program, Japanese Integrated Staging, and Okuda systems. However, our nomogram is focused on patients with end-stage HCC and PVTT who are unable to receive surgical resection, locoregional therapy, and/or sorafenib, based on the characteristics of the tumor and liver function.

Moreover, after stratifying the survival rates in the quartiles, we further identified the prognostic discrimination of the nomograms for the different BCLC stages and tumor diameters. All PVTT patients enrolled underwent conservative therapy. The 6-month OS of the low-risk group after stratification by nomogram score was significantly higher than the intermittent- and high-risk groups, especially in the BCLC C stage, tumor diameter ≥5 cm, and tumor diameter <5 cm subgroups. The nomogram can be used to evaluate the 1-, 3-, and 6-month prognosis of patients on the basis of available data on liver function and tumor stage, which will be helpful for explaining the disease stage to patients and their families. Moreover, patients in the low-risk group (0–85 scores) have 6-month overall survival rate of 83.3%, whose liver function and performance status can be actively monitored and it is possible to consider further active interventions to achieve the aim of prolonging survival such as TACE or molecularly targeted agents.

This is the first study to establish a nomogram for patients with HCC and PVTT on conservative treatment. Using this nomogram, we demonstrated that the 1-, 3-, and 6-month survival rates of patients with HCC plus PVTT were poor. We hope that the nomogram will assist in assessing prognosis for clinicians and help guide treatment decisions. The study has a few limitations. First, owing to the retrospective nature of the study, it is impossible to exclude the possibility of selection bias. However, we attempted to minimize this bias by performing multicenter research. Second, the suggested etiology of most of our patients was HBV-positive (83.7%). Although the proposed nomogram also provided a good c-index (0.707) for predicting OS in HBV-negative patients, its performance in patients with other etiologies remains to be investigated.

## 5. Conclusions

The nomogram established in this study may be useful for estimating the survival of HCC with PVTT on conservative treatment in a multicenter retrospective manner. After stratification by the nomogram score, the 6-month OS of the low-risk groups was significantly higher than that of the intermittent- and high-risk groups, which may help guide treatment decisions.

## Figures and Tables

**Table 1:** Clinical characteristics of patients with PVTT.

Variables	Derivation cohort *n* = 210 (%)	Validation cohort *n* = 102 (%)	*p* value
Median age (range)	54 (28–80)	62(34–91)	<0.001^a^
Sex (M/F)	177/33 (84.3/15.7)	97/5 (95.1/4.9)	0.006^b^
HBV related	190/20 (90.5/9.5)	71/31 (69.6/30.4)	<0.001^b^
Cirrhosis (yes/no)	193/17 (91.9/8.1)	94/8 (92.2/7.8)	0.939^b^
Comorbidity index^†^			<0.001^b^
0 score	131 (62.4)	85 (83.3)	
1 score	3 (1.4)	4 (3.9)	
2 score	68 (32.4)	8 (7.8)	
≥3 score	8 (3.8)	5 (4.9)	
ALT (IU/L) (≥70/<70)	74/136 (35.2/64.8)	19/83 (18.6/81.4)	0.003^b^
AST (IU/L) (≥119/<119)	78/132 (37.1/62.9)	26/76 (25.5/74.5)	0.041^b^
GGT (IU/L) (≥115/<115)	144/66 (68.6/31.4)	70/32 (68.6/31.4)	0.992^b^
Child–Pugh class (A or B/C)	176/34 (83.8/16.2)	78/24 (76.5/23.5)	0.118^b^
WBC (× 10^9^/L) (≥4.3/<4.3)	133/77 (63.3/36.7)	67/35 (65.7/34.3)	0.684^b^
Cr (*μ*moI/L) (≥91/<91)	28/182 (13.3/86.7)	19/83 (18.6/81.4)	0.220^b^
Portal vein invasion (Vp2-3/Vp4)	64/146 (30.5/69.5)	35/67 (34.3/65.7)	0.494^b^
AFP (ng/mL) (≥400/<400)	100/110 (47.6/52.4)	45/57 (44.1/55.9)	0.561^b^
Tumor number (≥3/<3)	121/89 (57.6/42.4)	46/56 (45.1/54.9)	0.038^b^
Tumor diameter (cm) (≥5/< 5)	130/80 (61.9/38.1)	59/43 (57.8/42.2)	0.491^b^
Lymph node metastasis (yes/no)	33/177 (15.7/84.3)	10/92 (9.8/90.2)	0.155^b^
BCLC stage (C/D)	168/42 (80/20)	86/16 (84.3/15.7)	0.358^b^
TNM stage (IIIB/IVA)	177/33 (84.3/15.7)	94/8 (92.2/7.8)	0.054^b^

Data are presented as *n* (%), or median (interquartile range). ^†^Comorbidity index includes 11 disease categories described fully in the Methods section. ^a^*t*-test. ^b^ Chi-square test or Fisher's exact test. HBV: hepatitis B virus; ALT: alanine transaminase; AST: aspartate transaminase; GGT: gamma-glutamyl transferase; WBC: white blood cell count; Cr: creatinine; AFP: *α*-fetoprotein; BCLC: Barcelona Clinic for Liver Cancer; TNM: tumor, node, metastasis staging.

**Table 2:** Univariate and multivariate Cox regression analyses for overall survival of HCC with PVTT in the derivation cohort.

Variable	Univariate Cox			Multivariate Cox		
	HR	95% CI	*p* value	HR	95% CI	*p* value
Median age	0.994	(0.975–1.013)	0.550			
Gender	0.936	(0.725–1.208)	0.610			
HBV related	1.130	(0.609–2.097)	0.699			
Cirrhosis	1.015	(0.776–1.327)	0.923			
Comorbidity index	0.980	(0.810–1.180)	0.802			
ALT ≥ 70 IU/L	2.006	(1.411–2.852)	<0.001			
AST ≥ 119 IU/L	2.849	(2.003–4.053)	<0.001	1.766	(1.194–2.611)	0.004
GGT ≥ 115 IU/L	2.679	(1.727–4.155)	<0.001	2.037	(1.265–3.279)	0.003
Child–Pugh class C	2.955	(1.958–4.459)	<0.001	3.294	(2.075–5.227)	<0.001
WBC ≥ 4.3 × 10^9^/L	2.075	(1.394–3.088)	<0.001			
Cr ≥ 91 *μ*moI/L	1.757	(1.099–2.808)	0.018	2.578	(1.573–4.223)	<0.001
Portal vein invasion Vp4	0.890	(0.613–1.290)	0.537			
AFP ≥ 400 ng/ml	1.900	(1.335–2.703)	<0.001	1.545	(1.073–2.227)	0.020
Tumor number ≥ 3	1.491	(1.041–2.135)	0.029			
Largest tumor diameter ≥ 5 cm	2.129	(1.436–3.157)	<0.001	2.784	(1.804–4.296)	<0.001
Lymph node metastasis	0.942	(0.579–1.532)	0.809			

HR, hazard ratios; CI, confidence interval.

## Data Availability

The clinical data used to support the findings of this study are restricted by the Ethics Committee of Beijing Ditan Hospital in order to protect patient privacy.

## References

[1] Chou R., Cuevas C., Fu R. (2014). *AHRQ Comparative Effectiveness Reviews. Imaging Techniques for the Diagnosis and Staging of Hepatocellular Carcinoma*.

[2] Chan S. L., Chong C. C., Chan A. W., Poon D. M., Chok K. S. (2016). Management of hepatocellular carcinoma with portal vein tumor thrombosis: review and update at 2016. *World Journal of Gastroenterology*.

[3] European Association for the Study of the Liver (2012). EASL-EORTC clinical practice guidelines: management of hepatocellular carcinoma. *Journal of Hepatology*.

[4] Llovet J. M., Ricci S., Mazzaferro V. (2008). Sorafenib in advanced hepatocellular carcinoma. *New England Journal of Medicine*.

[5] Tandon P., Garcia-Tsao G. (2009). Prognostic indicators in hepatocellular carcinoma: a systematic review of 72 studies. *Liver International*.

[6] Pirisi M., Avellini C., Fabris C. (1998). Portal vein thrombosis in hepatocellular carcinoma: age and sex distribution in an autopsy study. *Journal of Cancer Research and Clinical Oncology*.

[7] Hsieh C.-H., Wei C.-K., Yin W.-Y. (2015). Vascular invasion affects survival in early hepatocellular carcinoma. *Molecular and Clinical Oncology*.

[8] Sumie S., Nakashima O., Okuda K. (2014). The significance of classifying microvascular invasion in patients with hepatocellular carcinoma. *Annals of Surgical Oncology*.

[9] Katagiri S., Yamamoto M. (2014). Multidisciplinary treatments for hepatocellular carcinoma with major portal vein tumor thrombus. *Surgery Today*.

[10] Forner A., Llovet J. M., Bruix J. (2012). Hepatocellular carcinoma. *The Lancet*.

[11] Bruix J., Raoul J.-L., Sherman M. (2012). Efficacy and safety of sorafenib in patients with advanced hepatocellular carcinoma: subanalyses of a phase III trial. *Journal of Hepatology*.

[12] Chen M. Y., Wang Y. C., Wu T. H. (2016). Efficacy of external beam radiation-based treatment plus locoregional therapy for hepatocellular carcinoma associated with portal vein tumor thrombosis. *BioMed Research International*.

[13] Bruix J., Sherman M. (2011). Management of hepatocellular carcinoma: an update. *Hepatology*.

[14] Forner A., Reig M. E., Rodriguez de Lope C., Bruix J. (2010). Current strategy for staging and treatment: the BCLC update and future prospects. *Seminars in Liver Disease*.

[15] Llovet J., Brú C., Bruix J. (1999). Prognosis of hepatocellular carcinoma: the BCLC staging classification. *Seminars in Liver Disease*.

[16] Edge S. B., Compton C. C. (2010). The American Joint Committee on Cancer: the 7th edition of the AJCC cancer staging manual and the future of TNM. *Annals of Surgical Oncology*.

[17] Wan G., Gao F., Chen J. (2017). Nomogram prediction of individual prognosis of patients with hepatocellular carcinoma. *BMC Cancer*.

[18] European Association for the Study of the Liver (2012). EASL-EORTC clinical practice guidelines: management of hepatocellular carcinoma. *European Journal of Cancer*.

[19] Shah Z. K., McKernan M. G., Hahn P. F., Sahani D. V. (2007). Enhancing and expansile portal vein thrombosis: value in the diagnosis of hepatocellular carcinoma in patients with multiple hepatic lesions. *American Journal of Roentgenology*.

[20] Deyo R., Cherkin D. C., Ciol M. A. (1992). Adapting a clinical comorbidity index for use with ICD-9-CM administrative databases. *Journal of Clinical Epidemiology*.

[21] Cheung T.-K., Lai C.-L., Wong B. C.-Y., Fung J., Yuen M.-F. (2006). Clinical features, biochemical parameters, and virological profiles of patients with hepatocellular carcinoma in Hong Kong. *Alimentary Pharmacology & Therapeutics*.

[22] Yu J. I., Park H. C., Lim D. H. (2011). Prognostic index for portal vein tumor thrombosis in patients with hepatocellular carcinoma treated with radiation therapy. *Journal of Korean Medical Science*.

[23] Barman P. M., Sharma P., Krishnamurthy V. (2014). Predictors of mortality in patients with hepatocellular carcinoma undergoing transarterial chemoembolization. *Digestive Diseases and Sciences*.

[24] Su L., Zhang C., Zhao Y. (2014). Transarterial chemoembolization for the treatment of advanced hepatocellular carcinoma with portal vein tumor thrombosis: prognostic factors in a single-center study of 188 patients. *BioMed Research International*.

[25] Xiao J., Li G., Lin S. (2014). Prognostic factors of hepatocellular carcinoma patients treated by transarterial chemoembolization. *International Journal of Clinical and Experimental Pathology*.

[26] Zhang X.-B., Wang J.-H., Yan Z.-P., Qian S., Du S.-S., Zeng Z.-C. (2009). Hepatocellular carcinoma with main portal vein tumor thrombus. *Cancer*.

[27] Villa E., Moles A., Ferretti I. (2000). Natural history of inoperable hepatocellular carcinoma: estrogen receptors’ status in the tumor is the strongest prognostic factor for survival. *Hepatology*.

[28] Li S.-H., Wang Q.-X., Yang Z.-Y. (2017). Prognostic value of the neutrophil-to-lymphocyte ratio for hepatocellular carcinoma patients with portal/hepatic vein tumor thrombosis. *World Journal of Gastroenterology*.

[29] Liang H., Cui P., Guo Q. (2017). Prognostic factors of hepatocellular carcinoma patients with portal vein tumor thrombosis treated with transcatheter arterial chemoembolization. *Asia-Pacific Journal of Clinical Oncology*.

